# The therapeutic potential of *Salvia aegyptiaca* extracts in ethanol-induced gastric ulcer: insights into macroscopic, histopathological, and biochemical mechanisms

**DOI:** 10.55730/1300-0152.2722

**Published:** 2024-12-02

**Authors:** Walid MAMACHE, Smain AMIRA, Fatima BENCHIKH, Hassiba BENABDALLAH, Amor BENCHEIKH, Hind AMIRA, Roumaissa OUNIS, Mohammed Abdallah TORKI, Chahrazed KAOUDOUNE

**Affiliations:** 1 Laboratory of Applied Phytotherapy to Chronic Disease, Department of Animal Biology, Faculty of Natural Life Sciences, Ferhat Abbas University, Setif, Algeria; 2 Laboratory of Applied Microbiology, Department of Microbiology, Faculty of Natural Life Sciences, Ferhat Abbas University, Setif, Algeria

**Keywords:** *Salvia aegyptiaca*, gastric ulcer, lipid peroxidation, antioxidant enzyme

## Abstract

**Background/aim:**

This study explores the antiulcer activity of different doses of *Salvia aegyptiaca* (SAE) methanol (ME) and decocted extracts (DE) on ethanol-induced gastric ulcers in rats.

**Materials and methods:**

Female Wistar rats weighing 180–200 g were divided into five groups: control, omeprazole (positive control), and extract-treated (100, 200, and 400 mg/kg). Ulcers were induced with absolute ethanol 30 min after treatment with the extracts. The experiment was followed by macroscopic and histopathological examination. In vitro tests were also conducted to assess lipid peroxidation, catalase activity, mucus content, glutathione, and protein levels.

**Results:**

The study found that 100% ethanol caused significant damage, including colour and mucus loss, petechiae, haemorrhages, and oedema. However, pretreatment with ME SAE or DE SAE at doses of all three levels reduced the ethanol-induced damage. Histopathological analysis revealed reduced signs of haemorrhagic lesions, infiltration, and oedema in rats treated with ME SAE or DE SAE at doses of 100 or 200 mg/kg, whereas the 400 mg/kg dose provided complete protection. Comparable to the use of omeprazole, ingestion of DE SAE at doses of 100, 200, or 400 mg/kg demonstrated substantial protection against stomach ulcers produced by ethanol, with a range of 76%–84%. Both SAE extracts induced a dose-dependent increase in glutathione levels, with DE SAE showing a significant rise at 200 and 400 mg/kg.

**Conclusion:**

The SAE extracts demonstrated a significant decrease in gastric lipid peroxidation, outperforming the effect of omeprazole.

## 1. Introduction

Gastric ulcers (GU) are inflammatory disorders of the gastric mucosa caused by immune-mediated responses or pathogenic microorganisms that damage the stomach. The majority of cases of GU are observed on the lower curvature of the stomach and extend beyond the mucosal layer (Hassan et al., 2023). The most common symptom of GU is stomach pain; however, as a result of harm to the gastrointestinal epithelium and mucosa, GU can also induce other symptoms, such as abdominal discomfort, intense stomach pain, bloating, nausea, vomiting, and weight loss (Guzmán-Gómez et al., 2023). The causes of GU include *Helicobacter pylori* infection, alcohol consumption, and nonsteroidal anti-inflammatory drugs (NSAIDs) (Isnain et al., 2023). Estimates suggest a higher prevalence of this bacterium in low-income countries, affecting 80%–90% of young adults, compared to 40% or less in industrialized nations. In Algeria, the average prevalence of peptic ulcers is approximately 80% (Bachir et al., 2018). Inflammation, irritability, and cell loss in the stomach mucosa are symptoms of GU (Abdelfattah et al., 2019). Oxidative stress, inflammatory signalling, and apoptosis contribute to the aetiology of gastrointestinal illnesses, including GU. When inflammatory apoptosis begins, the caspase induction initiates a sequential series of reactions, resulting in irreversible apoptosis (Liu et al., 2023).

The general population is increasingly turning to natural treatments, such as honey, for GU (Elkordy et al., 2021). Recent research has demonstrated that honey possesses a variety of therapeutic advantages, including its antiinflammatory properties and its ability to prevent ulcers (Al-Ghamdi and Ansari, 2021). Moreover, numerous bioactive substances included in bee venom, such as peptides and enzymes, exhibit considerable efficacy in the treatment of inflammation and may serve as viable pharmaceutical options for alleviating pain (Lin and Hsieh, 2020). Recent years have also seen an increase in the usage of plants as an origin of pharmaceuticals due to their natural origin, accessibility in local communities, affordability, simplicity of use, and potential lack of complications (Abubakar and Haque, 2020). *Salvia aegyptiaca* (SAE), also referred to as Egyptian sage, has been observed to have numerous organic benefits. The plant contains powerful antioxidant compounds, consisting of flavonoids, phenolics, and tannins, that contribute to its antioxidant activity (Mamache et al., 2020; Nasr et al., 2023). Additionally, SAE extracts have shown antibacterial success against certain bacterial strains (Nasr et al., 2023), and the plant has been stated to possess anticancer properties, as its endophytic fungal microbiota are said to have anticancer benefits (El-Bondkly et al., 2020). SAE extracts have also been tested for their inhibitory effects on the enzymes involved in diabetes, including α-amylase and α-glucosidase (Mamache et al., 2020), and the plant has been used traditionally for its capability in treating diseases associated with the reproductive organs, sex hormones, infertility, and rheumatism (Tohamy et al., 2012). This study focused on the exploration of SAE as a natural remedy for ethanol-induced gastric ulcers, highlighting its significant antiulcer and antioxidant effects. By investigating the dose-dependent impacts of the methanolic extracts (ME) and decocted extracts (DE) of SAE, the research provides a detailed evaluation of its protective mechanisms, including enhanced antioxidant defence, reduced oxidative stress, and preservation of gastric mucus. The study demonstrates SAE’s comparable or superior efficacy to omeprazole, positioning it as a potentially sustainable and accessible alternative for ulcer treatment, particularly in regions like Algeria, where gastric ulcer prevalence is high. This work adds value by addressing local health challenges and advancing natural medicine research.

## 2. Material and methods

### 2.1. Plant material

*S. aegyptiaca* plants (Voucher number 104 SO 27/4/16 BAT/SA/HL) were harvested in May and June 2016. To ensure proper preservation, the plants were dried for 10 days in a shaded outdoor area, ensuring they were kept out of direct sunlight. The material was processed into a fine powder using an electric grinder (Molinex, France).

### 2.2. Preparation of extracts

The ME was prepared by macerating the plant powder at room temperature in 85% methanol at a rate of 15/100 (w/v) for 7 days. After filtration using muslin and filter paper, the filtrate was concentrated with a rotavapor (Buchi R-215, Switzerland) under vacuum at a temperature of 40 °C. The extracts obtained were then completely dried in an oven (Memmert, Germany) at 37 °C (Markham, 1982).

The DE was prepared by decocting the ME powder in distilled water according to Perera et al. (2008). A 30-g sample of ME powder was combined with 1L of distilled water and allowed to boil until the volume of the solution was reduced to 1/8th of the original volume. After filtration using muslin and filter paper, the filtrate was completely dried in an oven at 37 °C (Memmert, Germany).

### 2.3. Ethanol-induced GU in rats

The biological activities of the ME SAE and DE SAE were examined in vivo using female Wistar rats weighing 180–200 g that originated from the Pasteur Institute’s breeding facilities in Algiers. The experiment was conducted at the Setif animal facilities of Ferhat Abbas University. The rats were cared for in environments that supported their growth and development, with free access to ONAB (National Office for Livestock Feed) food and water. Before each experiment, the rats were housed individually in wire-mesh cages to prevent coprophagia. They were made to fast for 12–18 h but given adequate water up to 1 h before the experiment. The research conducted in this section was carried out following the ethical standards established by the Algerian Association of Experimental Animal Sciences (88-08/1988) and are aligned with the European Union Directive on the protection of animals used for scientific purposes (2010/63/EU). This commitment ensures that all experimental procedures are performed with consideration for animal welfare and ethical responsibility.

According to the modified protocol described by Benabdallah et al. (2022), The gastroprotective properties of ME SAE and DE SAE were tested using an ulcer induction model with absolute ethanol (Sigma). The rats were separated into five groups: a negative control given carboxy methyl cellulose (CMC), a positive control given omeprazole 5 mg/kg by mouth, and three extract-treated groups given doses of 100, 200, or 400 mg/kg by mouth. One hour after dosing, 0.5 mL 100% ethanol was administered orally, and each animal was euthanised by cervical dislocation within 30 min of being given the ethanol. Then, each stomach was removed, cut open across the great curvature, cleansed with NaCl, and positioned on a piece of corkboard to evaluate the surface of the ulcers. ImageJ v1.54d (NIH, USA) was used for the analysis, and the research results were represented as a percentage of protection.


$$\begin{array}{l} \%\;\text{ulceration}=\text{ulcerated surface}/\text{total surface }(\text{glandular part})\\ \%\;\text{protection}=(\%\;\text{ulcer control}/\%\;\text{ulcer treatment})\times 100 \end{array}$$

### 2.4. Preparation of homogenates

A portion of the glandular part of each stomach was homogenized in a Tris-HCl 50 mM (pH 7.4) buffer using a homogenizer. Each homogenate was centrifuged at 4000 g at 4 °C for 15 min (Sigma 3-30K, Germany). Then, the supernatant was collected and stored at −20 °C.

### 2.5. Preparation of histological slides

For classical histological studies, the preparation of thin sections for optical microscopy observation is carried out in several stages: sampling, fixation, postfixation, dehydration and circulation, inclusion (coating), sectioning, manipulation, colouring, final assembly and microscope observation. All of these steps were carried out in the Setif pathological anatomy laboratory.

The commonly used process for sample fixing is immersion in 10% formaldehyde for 24 h. For dehydration and circulation, the sample is first placed in alcohol baths of increasing concentrations from 50° to absolute alcohol of 100°, then in xylene or toluene baths for clarification, and then impregnated in paraffin at 58 °C for 24 h. This step prepares the sample for embedding, as paraffin is hydrophobic. In the inclusion (coating) stage, the samples in the melted paraffin are poured into a small metal mould (KARTELL LABWARE^®^ 2923, Italy) and heated to 56 °C. Once cooled, the paraffin solidifies into a block, which is is further processed by melting and sizing it to create a parallel-board cut pad. This prepared block is then passed through a microtome, which slices it into thin sections of 5 μm. These sections are systematically arranged into strips. Finally, the sections are mounted onto glass slides. The next step was to soften the paraffin by placing the slides on a plate heated to 45–60 °C for 15 min. The paraffin was removed by immersing the slides in toluene or xylene baths, and then rehydration was done by immersing the slides in alcohol baths of decreasing concentrations from 100° to 50°, ending with a distilled water bath. The The staining process followed a specific sequence: xylene → alcohol → water → emalon → water → eosin → water → water → alcohol. After staining, the thin sections underwent another round of dehydration. This was achieved by passing them through a series of alcohol baths with progressively increasing concentrations, followed by toluene baths. Once dehydrated, the stained thin sections were mounted between a glass slide and a cover slip using a synthetic resin. The resin used had a refractive index similar to that of glass, ensuring optimal clarity for microscopic observation.

### 2.6. Determination of total gastric protein

Total gastric protein concentration was measured using the technique of Gornall et al. (1949) using a Bradford reagent. Absorbance (Abs) was measured using a UV-Vis spectrophotometer (Shimadzu UV1800). The results are presented as μg/mg of tissue. The total protein concentration was calculated using the following equation in which n is the standard concentration:


Total proteins (mg/mL)=(Abs test/Abs standard)×n

### 2.7. Determination of glutathione

The measurement of reduced glutathione (GSH) content was done using Ellman’s procedure (1959). The concentration of GSH is calculated using the molar absorption coefficient (e NBT: 13.6 × 103 M^−1^cm^−1^). The results are presented as nmol of GSH/g of tissue.

### 2.8. Estimation of lipid peroxidation

The gastric tissue lipid peroxidation was estimated by measuring the content of malondialdehyde (MDA) using the method described by Ohkawa et al. (1979). The concentration of MDA is calculated using the molar absorption coefficient (MDATBA: 156 mM^−1^cm^−1^). The results are presented as nmol of MDA/g of tissue.

### 2.9. Determination of catalase activity

Catalase (CAT) activity was measured using the method described by Clairbone (1985) with some modifications. The principle of this test is based on the breakdown of hydrogen peroxide in the presence of CAT. The enzymatic activity is presented as nmol H_2_O_2_/min/mg.

### 2.10. Statistical analysis

The results were presented as mean or average ± SEM and were subjected to analysis of variance followed by Tukey’s test for multiple comparisons using GraphPad Prism (GraphPad Software, Inc., USA). A significance level of p < 0.05 or lower was deemed be a statistically significant difference.

## 3. Results

### 3.1. The effect of SAE on macroscopic and histopathological appearance

**Figure 1:**
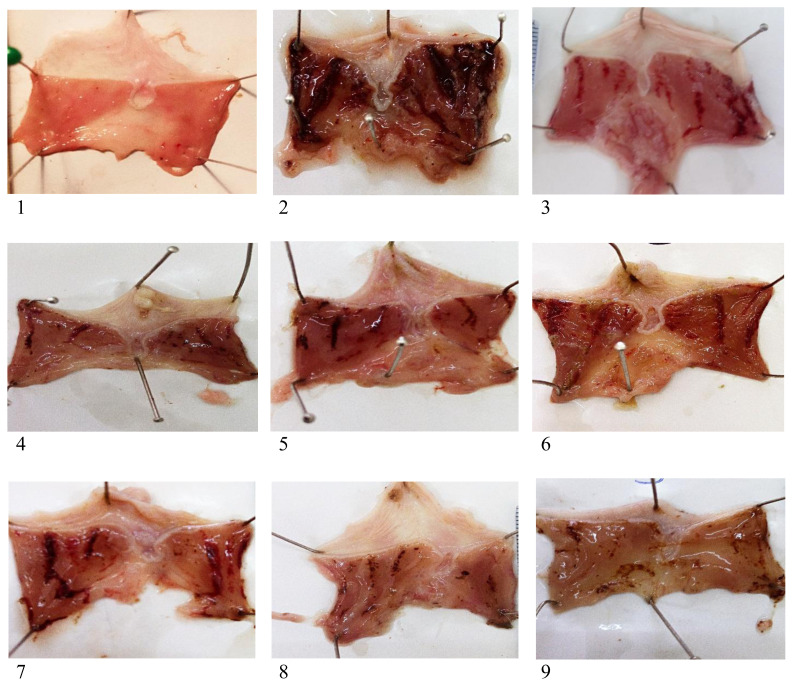
The effect of SAE on the visual characteristics of the stomach mucosa in gastric ulcers caused by ethanol. 1: control, 2: ethanol 100%, 3: omeprazole 5 mg/kg, (4–6): ME SAE at 100, 200, and 400 mg/kg doses, respectively, (7–9): DE SAE at 100, 200, and 400 mg/kg doses, respectively.

**Figure 2:**
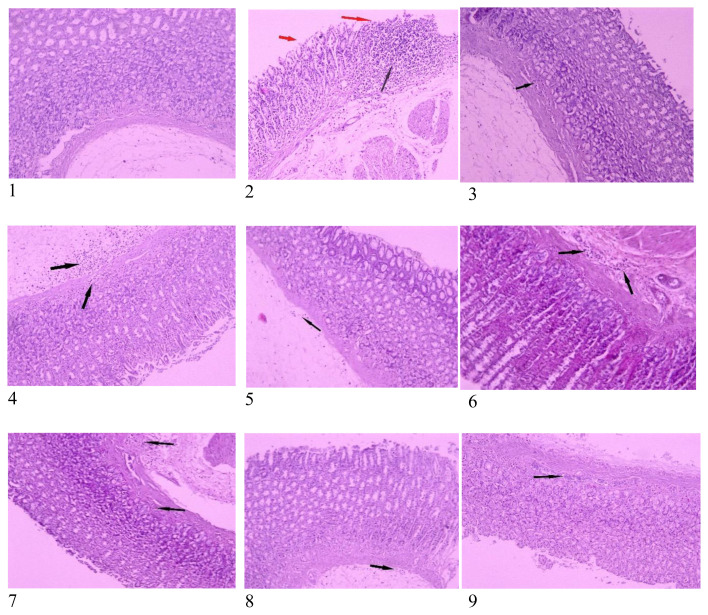
Histological assessment of the preventive effect of SAE against ethanol-induced GU at 100× magnification. Red arrows indicate damage to the epithelial barrier, and black arrows indicate infiltration of inflammatory cells.

Visual examination revealed a notable impact of the ethanol on the gastric tissues (Figure 1). Rats exposed to 100% ethanol exhibited uniform and noticeable injuries that were evidenced by loss of normal colour and mucus as well as the presence of petechiae, haemorrhages, and oedema. However, this damage was mitigated by the administration of ME SAE or DE SAE at different doses. Pretreatment of rats with ME SAE or DE SAE at doses of 100, 200, and 400 mg/kg reduced the ethanol-induced damage (Figure 1). These results were supported by the histopathological analysis. There are fewer signs of haemorrhagic lesions, less infiltration, and less oedema in the gastric mucosa of the rats treated with DE SAE and ME SAE at 100 and 200 mg/kg. Rats receiving the highest dose of 400 mg/kg were fully protected from the ethanol’s effects and maintained all histological characteristics compared to the control group (Figure 2).

### 3.2. The effect of DE SAE on ethanol-induced GU

**Figure 3:**
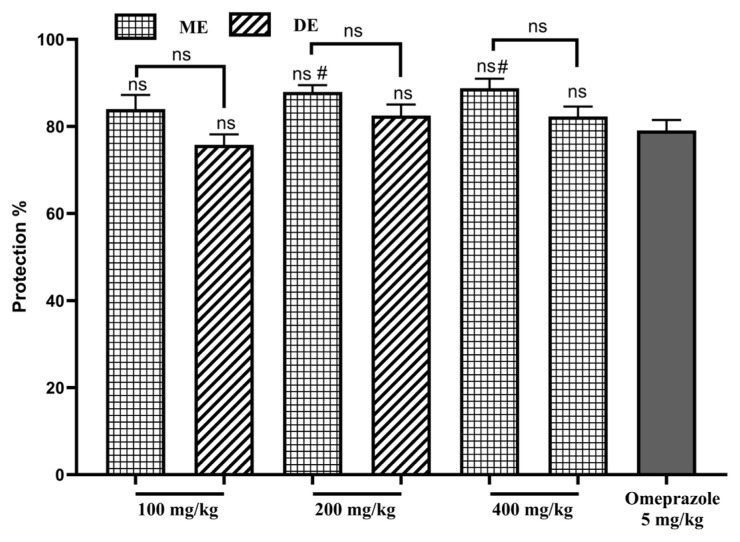
The impact of SAE on the stomach mucosa in ethanol-induced GU. ME: methanolic extract, DE: decocted extract. Results are represented as average % ± SEM, # indicates p ≤ 0.05 vs omeprazole, and ns means not significant.

Ingestion of 100, 200 and 400 mg/kg DE SAE provided significant protection in the range of 76%–84%, comparable to that of omeprazole (79%, p > 0.05, Figure 3). However, significant (p ≤ 0.05) protection levels of 87.98% and 88.75% were detected following the use of 400 and 200 mg/kg ME SAE, respectively. There are no significant differences between the types of extracts.

### 3.3. Effect on total gastric mucus content

**Figure 4:**
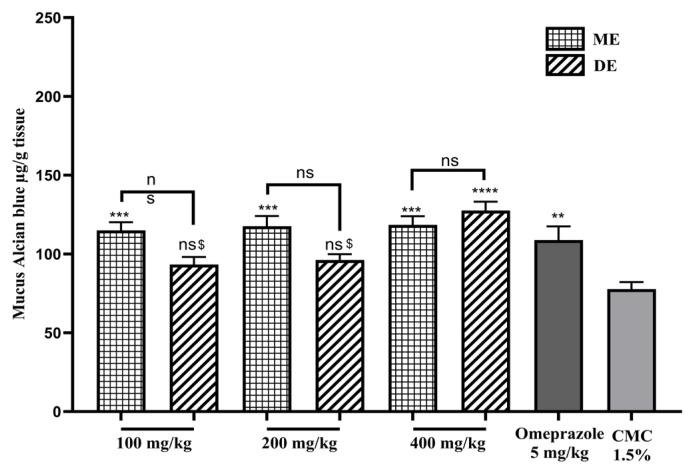
The impact of SAE on stomach mucus content. ME: methanolic extract, DE: decocted extract. Results are represented as average % ± SEM. ** indicates p < 0.001, and *** indicates p < 0.002 vs. CMC 1.5%. $ indicates p ≤ 0.05 vs. 400 mg/kg, and ns means not significant.

The effect of SAE on gastric mucus content is shown in Figure 4. Compared to the group of animals treated with CMC 1.5% alone, ME SAE significantly (p ≤ 0.0002) increased gastric mucus levels for all doses used. On the other hand, DE SAE only showed an increase (p ≤ 0.0001) for the 400 mg/kg dose, which is only slightly greater than that of omeprazole (Figure 4).

### 3.4. Effect on protein content

**Figure 5:**
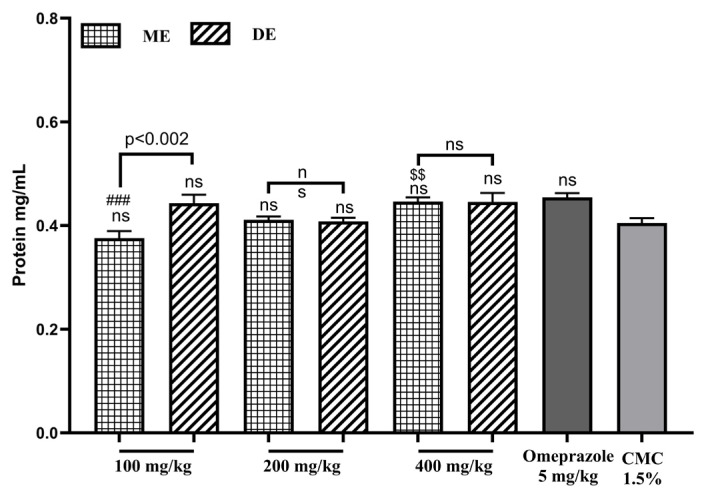
The impact of SAE on protein concentration. ME: methanolic extract, DE: decocted extract. Results are represented as an average % ± SEM. $$ indicates p ≤ 0.001 vs. 100 mg/kg, ### indicates p ≤ 0.0002 vs. omeprazole, and ns means not significant.

The treatment with the different extract doses had no effect on protein content (p > 0.05) except in the 400 mg.kg^−1^ SAE ME treated groups (Figure 5).

### 3.5. Effect on catalase activity

**Figure 6:**
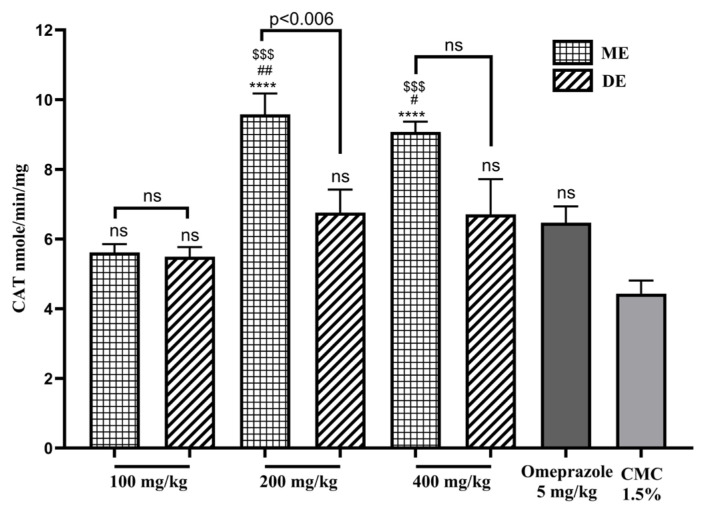
The effect of SAE on CAT activity. ME: methanolic extract, DE: decocted extract. Results are represented as average % ± SEM. **** indicates p ≤ 0.0001 vs. CMC 1.5%, $$$ indicates p ≤ 0.0002 vs. 100 mg/kg, # indicates p ≤ 0.05 vs. omeprazole, and ns means not significant.

The treatment of rats with ME SAE induced an increase in CAT activity, which was significant (p < 0.0001) for doses 200 and 400 mg/kg; the CAT activity for these doses was 9.58 ± 0.6 and 9.08 ± 0.3 (n = 8) nmol/min/mg, respectively. In contrast, the CAT activity after the DE SAE doses was comparable to that of omeprazole (Figure 6).

### 3.6. Effect on GSH level

**Figure 7:**
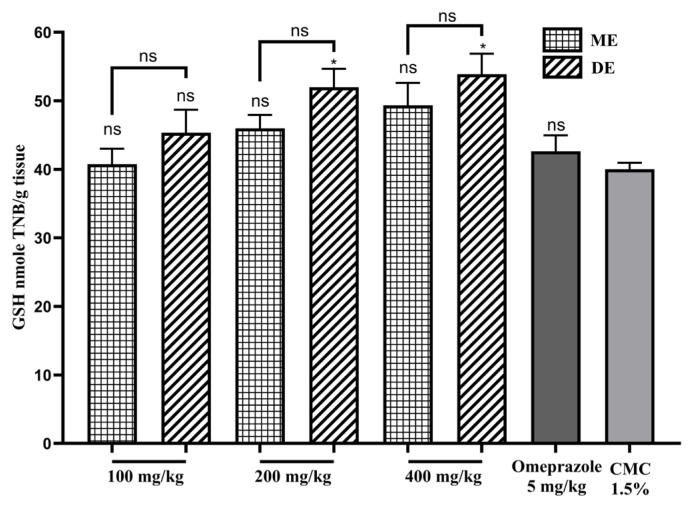
The effect of SAE on GSH level. ME: methanolic extract, DE: decocted extract. Results are represented as average % ± SEM. * indicates p ≤ 0.05 vs. CMC 1.5%, and ns means not significant.

The treatment of rats with SAE extracts induced a dose-dependent increase in GSH levels. This increase was significant (p ≤ 0.05) for the 400 or 200 mg/kg doses of DE SAE only. In this case, the GSH values are 52.0 ± 2.67 and 53.89 ± 3.01 nmole TNB/g, respectively. The GSH levels observed with the ME SAE doses were only slightly elevated compared to that of omeprazole (42.65 ± 2.34 nmole TNB/g, Figure 7).

### 3.7. Effect on lipid peroxidation

**Figure 8:**
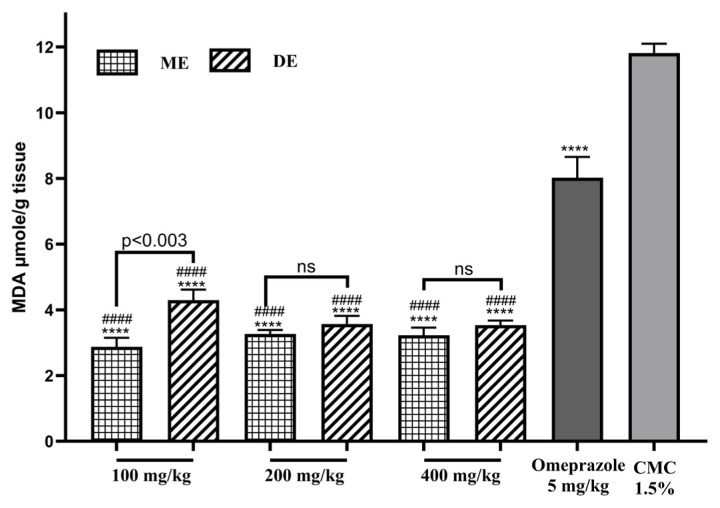
The effect of SAE on lipid peroxidation. ME: methanolic extract, DE: decocted extract. Results are represented as average % ± SEM. **** indicates p ≤ 0.0001 vs CMC 1.5%, #### indicates p ≤ 0.05 vs. omeprazole, and ns means not significant.

Lipid peroxidation and MDA content increased following ethanol application. In the case of pretreatment with omeprazole, the MDA content decreased (p ≤ 0.0001) by 08.02 ± 0.63 μmol/g tissue. Pretreatment with different doses of ME SAE or DE SAE decreased gastric lipid peroxidation (p ≤ 0.0001), and the rates of MDA decrease varied between 7.52 and 8.94 μmol/g (Figure 8). All SAE doses showed a significant effect compared to omeprazole.

## 4. Discussion

The induction of GU is done using ethanol because it can quickly penetrate the stomach lining after a few minutes of ingestion, causing haemorrhagic sores and tissue damage due to the cessation of blood flow to the stomach (Arab et al., 2015). Ethanol can also damage the cell plasma membrane, thereby increasing the vascular permeability of sodium and water, the accumulation of calcium inside the mucosa, and the destruction of the epithelial surface (Miller et al., 2001). Ethanol triggers oxidative stress by promoting the generation of the superoxide anion and hydroxyl radical, inducing peroxidation of gastric lipids as well as ulceration (Nyam et al., 2016). Ethanol can also induce gastric mucosal damage by stimulating the formation of leukotrienes. This study demonstrates that the introduction of ethanol to rats results in significant damage to the gastrointestinal tissue, characterised by the presence of petechiae, haemorrhage, and oedema. These lesions are likely caused by the depletion of mucus from the veins and arteries in the gastric mucosa, resulting in haemorrhage, inflammation, and tissue injury. To determine the antiulcer properties of SAE, an assessment of stomach tissues was conducted using histological examination. Microscopic examination of the stomachs of rats exposed to ethanol revealed histopathological alterations in the gastric tissues. Ethanol-induced histopathological damage includes separation of the surface epithelium, swelling, bleeding, stomach ulcers, and an inflammatory response marked by neutrophil infiltration. Treatment with SEA inhibited the effects of the ethanol by preserving the gastric wall.

Oxidative stress plays a key role in ethanol-induced GU by promoting the generation of reactive oxygen species and inducing lipid peroxidation, which leads to cellular damage and inflammation. Pretreatment with SAE extracts significantly increased gastric mucus content, highlighting their protective effects. Similar findings have been reported with the Tingli Dazao Xiefei decoction, which ameliorates inflammation, oxidative stress, and cellular damage through the modulation of NO–CO metabolic pathways and immune balance (Ruan et al., 2023). Other possible protective effects of these plants may involve pathways, such as the cyclooxygenase and nitrergic, by activation of COX and NOS, along with inhibiting gastric secretion and enhancing mucosal blood flow (Khattab et al., 2001).

The results of other studies have demonstrated that the different extracts or fractions prepared from the plants *S. officinalis* L and *S. plebeia* can significantly reduce ethanol lesions in rats (Nugroho et al., 2012; Fiorentin et al., 2013). The gastroprotective effect of the ethanolic extract of *Solenostemon monostachyus*, the aqueous extract of *Pseuderanthemum palatiferum*, and the hydroalcoholic extract of *Nigella sativa* seeds may be related to their suppression of lipoxygenase activity and/or their activation of gastric mucus secretion (Paseban et al., 2020).

Proteins, especially albumin, are considered extracellular antioxidants, an important type of secondary antioxidant that binds iron, copper, and many products such as hydroperoxides, preventing free radical-induced reactions in the cell membranes or at the protein level to protect the cells from destruction (Migdal and Serres, 2011). To assess oxidative stress in rats with ethanol-induced GU, various oxidant–antioxidant characteristics were examined. Ethanol-induced GU results in significant oxidative stress, as demonstrated by the elevated levels of lipid oxidation and decreased levels of intracellular antioxidants such as GSH and CAT. However, the administration of different doses of SAE caused an increase in CAT activity, GSH content, and a sharp decrease in gastric lipid peroxidation, suggesting that its antioxidant properties contribute to the stabilization of cellular membranes and prevention of oxidative damage in gastric tissues. Similarly, studies on metabolic disorders, such as on the impact of microcystin-LR on liver lipid metabolism via the PI3K/AKT/mTOR/SREBP1 signalling pathway, demonstrate the critical role of oxidative stress and lipid regulation in pathological conditions (Chu et al., 2022). These findings underscore the interconnected nature of oxidative stress, lipid metabolism, and inflammation in tissue repair. In addition to their ulcer-healing potential, plant-derived compounds are increasingly recognized for their anticancer properties. For instance, celastrol has been shown to inhibit gastric cancer cell proliferation, migration, and invasion (Peng et al., 2023). Studies have evaluated the antiulcerogenic activity and mechanisms of *Indigo feratruxillensis*, *Salvadora pericia*, and *Rosmarinus officinalis* leaves against ethanol-induced ulcers in rats, and found that they promoted a significant increase in gastric GSH content (Luiz-Ferreira et al., 2012; Amaral et al., 2013; Lebda et al., 2018). As GSH is an endogenous antioxidant that is considered the first line of defence against free radicals, a cell with a high GSH level makes its components less susceptible to oxidation (Anfal and Sahib, 2015).

The development of GU is influenced by multiple factors, with the secretion of stomach acid being widely acknowledged as a key element in this condition. Therefore, the primary focus of therapy is to regulate this secretion through the use of antisecretory medications. Multiple studies have demonstrated the ability of plant extracts to reduce secretion in experimental ulcer models (Mayer et al., 2009; da Silva Jr et al., 2016). For many plants, their therapeutic characteristics are mostly ascribed to the existence of phenolic acids and flavonoids; however, other substances, such as alkaloids and terpenoids, could also be therapeutically beneficial (Bonamin et al., 2014; Zhang et al., 2014). Phenolic chemicals, particularly flavonoids, have experimentally been found to shield the gastrointestinal mucosa against the harm induced by many ulcer types. Quercetin possesses antihistamine characteristics, which means it hinders the secretion of histamine from stomach mast cells and blocks the activity of the H^+^/K^+^ gastric proton pump, resulting in a decrease in the secretion of stomach acid (Kahraman et al., 2003). Other flavonoids, specifically chalcones and their derivatives, are accountable for several biological functions, including enhanced blood flow in the mucosal lining, greater gastric mucus production, elevated levels of prostaglandins, superoxide dismutase, and glutathione peroxidase activities, and a significant reduction in MDA (Dhiyaaldeen et al., 2014; Mohan et al., 2020). However, the main reason for the significant antiulcer activity shown by flavonoids is their remarkable antioxidant capacity. Quercetin and rutin, which are free radical scavengers and inhibitors of oxidative enzymes, are responsible for the chelation of transition metal ions and lipid peroxidation inhibitors (Song et al., 2018). Studies have concluded that carnosate and carnosol are responsible for antioxidant activity and have antiinflammatory properties, thereby protecting the stomach against gastric damage and reducing stomach acid secretion (Fiorentin et al., 2013).

Caffeic acid, luteolin, and rosmarinic acid, major compounds in the studied plants of the Lamiaceae family and Salvia genus, are known for their antiulcer activity (Kangwan et al., 2019). Ingestion of 200 mg/kg of caffeic acid greatly reduced ethanol-induced lesions in the gastric mucosa of rats. This effect has been attributed to its antioxidant properties (Okada et al., 2005). The mechanism of action of rosmarinic acid involves several parameters, including the maintenance of the gastric mucosal barrier through its interaction with sulfhydryl groups (do Nascimento et al., 2020). PGs are present throughout the gastrointestinal canal and are involved in the regulation of various gastric functions that protect the gastric mucosa from aggressive agents, stress, and nonsteroidal antiinflammatory drugs by controlling acid secretion, mucus production, and increasing mucosal blood flow (Miller 1983; Takeuchi and Amagase, 2017). From this effect, luteolin-7-O-glucoside and rosmarinic acid increase the production of beneficial prostaglandins E2, a mechanism that may be involved in the protection of GU (Al-Sereiti et al., 1999; Antonisamy et al., 2016). However, a recent study excludes the involvement of PG production by rosmarinic acid in its protective mechanism (Kangwan et al., 2019). One of the important mechanisms involved in GU protection is the inhibition of inflammation. Luteolin and rosmarinic acid are known as inhibitors of inflammation in gastric cells via inhibition of neutrophil infiltration, myeloperoxidase, cyclooxygenase 1 and 2 activity, and proinflammatory cytokines such as IL1, IL6, IL10, and TNFα (Antonisamy et al., 2016; Kangwan et al., 2019; do Nascimento et al., 2020). The significant antioxidant activity of rosmarinic acid and luteolin is closely associated with their gastroprotective effects; the latter is characterised by an increase in the action of endogenous antioxidant enzymes, the content of intracellular GSH, and a sharp reduction in lipid peroxidation (Antonisamy et al., 2016; Deger and Çavus, 2020).

## 5. Conclusion

Both ME SAE and DE SAE protected the stomach from ethanol-induced GU. This protection was expressed in vitro by increased gastric mucus content, preserved protein levels, increased GSH content, decreased lipid peroxidation, and increased activity of antioxidant enzymes such as CAT.
